# Development of the International Classification of Functioning, Disability and Health Core Set for Vision Loss: Insights From the Lived Experience of People With Vision Loss

**DOI:** 10.1007/s44402-026-00031-5

**Published:** 2026-03-06

**Authors:** Lorenzo Billiet, Hilde P. A. van der Aa, Dominique Van de Velde, Ferhat Esatbeyoglu, Raba Thapa, Vijaya K. Gothwal, Stijn De Baets, Ruth M. A. van Nispen

**Affiliations:** 1 Department of Ophthalmology, Amsterdam UMC, location Vrije Universiteit, Amsterdam, The Netherlands; 2 Quality of Care, Amsterdam Public Health, Amsterdam, The Netherlands; 3 Department of Rehabilitation Sciences - Occupational Therapy Research Group, Faculty of Medicine and Health Sciences, Ghent University, Ghent, Belgium; 4 Blindenzorg Licht en Liefde, Varsenare, Belgium; 5 The Lighthouse Guild, New York, NY USA; 6 Department of Physical Education and Sport Teaching, Faculty of Sport Sciences, Yozgat Bozok University, Yozgat, Turkey; 7 Tilganga Institute of Ophthalmology, Kathmandu, Nepal; 8 Meera and L B Deshpande Centre for Sight Enhancement, Institute for Vision Rehabilitation, Hyderabad, India; 9 Faculty of Medicine and Pharmacy, Vrije Universiteit Brussel, Brussels, Belgium

**Keywords:** Core Set, Disability and health, International Classification of Functioning, Low vision, Patient perspective, Vision impairment

## Abstract

**Objective:**

The global increase in life expectancy has led to more visual impairment, significantly impacting the quality of life and daily activities of individuals living across the world’s continents. This study aims to develop an ICF Core Set Vision Loss for the adult life span, providing a global perspective on the functional challenges faced by individuals with vision impairment. As part of its development, the aim was to understand the impact of vision loss on the lives and functioning of individuals with vision impairment from their own perspectives.

**Method:**

A cross-sectional qualitative study was performed, using a thematic analysis of data that was gathered through an online survey across the six World Health Organization regions and in-depth interviews. Meaningful concepts were linked to the ICF categories based on available linking rules.

**Results:**

Analyses of the online surveys with 663 participants in 59 countries and 100 in-depth interviews in Nepal and India resulted in 7652 meaningful concepts that could be linked to 148 ICF categories: 9% related to body functions and structures, 49% to activities and participation and 42% to environmental factors. Key findings regarding body functions and structures included a focus on emotional functions, confidence and energy levels. For activities and participation, major concerns were recreation, transportation and employment. Environmental factors highlighted the importance of family and technology.

**Conclusion:**

This study underscores the complex challenges faced by individuals with vision loss, encompassing emotional, psychological, environmental and participation-related aspects. These insights highlight the need for tailored assessments, interventions and comprehensive support systems. By comparing these findings with those from other preparatory studies, these results contribute to a deeper understanding of the lived experience of vision loss and provide an essential step in the future development of the ICF Core Set for Vision Loss.

Key Points

- Differences between World Health Organization regions reveal varying priorities: mental health concerns were emphasised in Europe and Southeast Asia, while social support dominated in Africa, highlighting cultural and contextual influences on functioning.
- This study shows that vision loss affects multiple life domains beyond sight, including mobility, employment, recreation and emotional well-being, underscoring the need for holistic and tailored interventions.
- Family support and assistive technology emerged as universal enablers of independence, indicating that strengthening social networks and access to technology can significantly improve quality of life globally.

## Introduction

Due to the global increase in life expectancy, ageing has become a dominant characteristic of the adult life span. As a result, a growing proportion of the adult population is affected by chronic conditions, illnesses and disabilities, including visual impairment. Although the age-standardised prevalence of visual impairment is declining globally, the absolute number of people with visual impairment is rising. This increase is primarily driven by population growth and ageing, as highlighted by Bourne et al. [1]. In 2020, it was estimated that 43 million people worldwide were blind, 295 million people had moderate to severe visual impairment and 258 million people had mild visual impairment [1–3].

The International Classification of Functioning, Disability and Health (ICF) provides a comprehensive framework for understanding participation challenges experienced by individuals with visual impairments. The ICF framework identifies key domains, including body functions, activities, participation and environmental factors that influence self-perceived health in this population [4]. Living with visual impairment significantly impacts an individual’s functioning. Evidence shows that visual impairment negatively affects people’s quality of life [5] and the performance of activities of daily living [6, 7].

The ICF framework highlights both individual and societal barriers to participation. Especially, individuals with more severe visual impairments and recent sight loss often face greater individual barriers [8]. Studies have shown that people with visual impairment participate less in household, recreational and sports activities compared to their peers [9]. Young visually impaired individuals encounter specific challenges in mobility, domestic life, interpersonal relationships and leisure activities [10]. Social engagement often requires the ability to perform visually demanding tasks [7], posing additional difficulties [11]. Individuals with visual impairment are at risk of disability, poor health, unemployment, low financial income and adverse interpersonal events [12]. Challenges in forming and maintaining social relationships can further limit employment opportunities [13]. These difficulties affect individuals across all age groups, leading to feelings of loneliness [14] and increasing the risk of anxiety, depression [15] and fatigue [16]. Additionally, older individuals with visual impairments face other risks, such as injuries [17] and falls [18–20]. These findings highlight the importance of comprehensive, individualised assessments of participation challenges for individuals with visual impairment.

Due to the comprehensive nature of the ICF, different Core Sets have been developed [21, 22]. An ICF Core Set is a customised list of ICF categories that describe the functioning and disability of a particular population. It includes essential categories from the full ICF classification, deemed most relevant for assessing the functioning of individuals within a specific healthcare context. An ICF Core Set Vision Loss in young and older adults potentially offers a framework to evaluate various aspects of functioning, providing a holistic view of an individual’s functional level that may be affected by vision loss.

The overall aim is to develop an ICF Core Set Vision Loss. Selb et al. outlined a methodology [23] which has been applied in other Core Set developments, such as those for hearing loss [24] and dual sensory loss [25, 26]. This methodology was specified for the ICF Core Set Vision Loss and also aimed to follow the various phases [27]. As a first step in the preparatory phase, the perspective of researchers was mapped through a systematic review of studied topics and questionnaires [28] and subsequently gained an in-depth local insight via focus groups [29]. As a next step, the current study aimed to provide important functional and daily challenges of individuals with vision loss from their own perspectives, who live across the six World Health Organisation (WHO) regions.

## Method

The study received ethical approval from the Ethical Committee of Ghent University Hospital, Belgium (B6702021000329), Amsterdam UMC, Netherlands (IRB00002991), Hyderabad Eye Institute, India (BHR-P-07-23-1075), Nepal Health Research Council (748/2023) and was conducted in accordance with the principles outlined in the Declaration of Helsinki.

### Study Design

A cross-sectional qualitative study was performed by using a thematic analysis of the data obtained from online surveys and individual structured interviews. The online survey was available in 11 languages (English, Spanish, Dutch, French, Portuguese, Italian, Turkish, Arabic, Ukrainian, Russian and German). For the individual interviews, there was a collaboration with researchers from the Hyderabad Eye Institute in India and the Tilganga Institute of Ophthalmology in Nepal, who conducted interviews in Hindi and Nepali. This approach comprehensively explored the impact of vision loss on daily life functioning.

### Participants, Sampling Method

Participants were recruited from across all WHO regions [30]. Study participants were selected by a quota sampling with a maximum variation strategy [31], to ensure representation across all six WHO regions, age, sex and severity of visual impairment.

Recruitment procedures varied according to the local context, ranging from identification through client databases of rehabilitation centres, snowball sampling with participants spreading the call to participate to others, or referrals through professionals or service providers.

### Study Population

Individuals living with visual impairment were included as defined by the WHO [32] as the target population. Persons with vision loss were considered eligible for participation in the surveys or interviews if they (1) self-identified as visually impaired or blind, regardless of clinical confirmation, (2) if they were willing to openly discuss and share their personal experiences on various aspects related to functioning and the contextual factors that impacted their vision loss, (3) if they were fluent in one of the languages in which the surveys or the interviews were available, (4) if they were at least 18 years old and (5) if there were no other conditions present that could impact their daily functioning. This was assessed with various questions within the survey and before conducting the interviews. Participants were not compensated for taking part in the study.

### Data Collection

Participants provided consent for the online surveys by agreeing to an informed consent form prior to starting the survey. For the individual interviews, consent was obtained face-to-face before the interview, after having received written and oral information about the study purposes.

The online survey consisted of two parts. In the first part, demographic variables of the participants were collected. In the second part, thirteen open-ended questions were asked to address the different chapters of the ICF, similar to previously developed Core Sets [33–36]. The questions followed the ICF components: two questions about body functions and structures, four about environmental factors and seven about activity and participation. Personal factors were not asked since these are not categorised in the ICF. The structured interview followed the same question set as used in the online survey, without any additional probing or follow-up questions.

When necessary, transcripts of answers conducted in languages other than English, Dutch, Turkish, Hindi and Nepali were translated using DeepL Translator Software (deepl.com)®.

### Data Analysis

To analyse the cross-sectional data, Braun and Clarke’s [37] guidelines for thematic analysis were followed. In the first phase, the transcripts of the online surveys and individual interviews were thoroughly read, and in vivo coding was applied independently by two junior researchers under the guidance of the first author. The first author, with a background in ICF and ophthalmology, trained the junior researchers, who then analysed the first 50 surveys, received feedback and continued with the analysis.

In the second phase, agreement on the in vivo codes was reached, and similar codes were grouped together and labelled with more abstract open codes. The third phase involved further revision, refinement, comparison and rephrasing the codes into categories. In the fourth phase, constant comparison and discussion between the three researchers took place to identify the themes. To carry out this process, ATLAS.ti software (atlasti.com)® was used.

Subsequently, the relevant concepts were linked to the ICF framework. Each meaningful concept was linked to a single ICF category, using the linking rules by Cieza et al. [38]. Key points of the linking rules included: (1) a meaningful concept was linked to the most precise ICF category; (2) code 8 (other specified) or 9 (unspecified) were only used when there were no other options available (the code not definable (nd) was not used); (3) only one category was chosen to link a meaningful concept and (4) if a meaningful concept was repeatedly mentioned within the survey of a single participant, it was assigned only once to an ICF category. The methodology for data linking in this qualitative study followed the same procedures as those implemented in the earlier ICF systematic literature review conducted in this ongoing research [28].

## Results

### Participants Characteristics

**Fig. 1:**
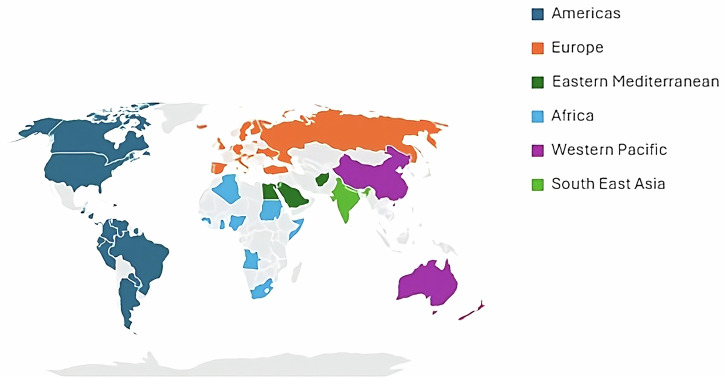
Global distribution of countries across the six WHO regions. This map displays the geographic classification of Member States according to the six World Health Organization (WHO) regions. Countries are colour‑coded as follows: Americas (dark blue), Europe (orange), Eastern Mediterranean (green), Africa (light blue), Western Pacific (purple) and Southeast Asia (light green). The coloured shading highlights regional boundaries and facilitates visual comparison of regional groupings across the globe.

The online survey and structured interviews included 763 participants (663 surveys and 100 interviews) from 61 countries across all 6 WHO regions, with Europe (*n* = 505; 66%) and Southeast Asia (*n* = 119; 16%) emerging as the largest contributors (Americas *n* = 71 participants, *n* = 14 from the Eastern Mediterranean, *n* = 33 from the Western Pacific and *n* = 20 from Africa). Among all participants, 39% (*n* = 296) reported being blind. The average age of the participants was 54 years. The Eastern Mediterranean region was characterised by a significantly younger population, with an average age of 32.8 years and a predominantly male demographic (71.4%) relative to other WHO regions. In the African region, 81% of participants were male, while in Southeast Asia, 31% of participants had only primary education (Table 1 and Fig. 1).

**Table 1:** Socio-demographic variables of study participants in the surveys or interviews (*N* = 763).

Characteristics	Americas *N* = 71	Eastern Mediterranean *N* = 14	Europe *N* = 505	Southeast Asia *N* = 119	Western Pacific *N* = 33	Africa *N* = 21	Total all regions *N* = 763
Mean age, SD [range]	50.4 ± 12.5 [23–65]	32.8 ± 13.1 [18–63]	52.2 ± 9.95 [18–53]	45.2 ± 11.5 [18–56]	5451 ± 19.5 [24–87]	46 ± 20.2 [18–100]	54.5 ± 22.1 [18–100]
Sex *n* [%] (Male)	29 [40.8]	10 [71.4]	253 [50.1]	76 [63.9]	16 [48.5]	17 [81]	322 [42.2]
Education level *N* (%)							
PhD	6 (8)	1 (7)	14 (3)	2 (2)	2 (6)	0	25 (3)
Master	18 (25)	4 (29)	147 (29)	13 (11)	3 (9)	4 (19)	189 (25)
Bachelor	21 (30)	5 (36)	80 (16)	36 (30)	8 (24)	5 (24)	155 (20)
High school	23 (32)	3 (21)	207 (41)	31 (26)	13 (39)	8 (38)	285 (37)
Primary education	1 (1)	0	36 (7)	24 (20)	4 (12)	0	65 (9)
No diploma	2 (3)	1 (7)	21 (4)	13 (11)	3 (9)	4 (19)	44 (6)
Marital status *N* (%)							
Divorced	5 (7)	0	41 (8)	0	2 (6)	0	48 (6)
Living together	3 (4)	0	32 (4)	0	3 (6)	0	39 (5)
Married	25 (35)	3 (21)	225 (45)	64 (54)	17 (52)	10 (48)	344 (45)
Single	34 (48)	11 (79)	172 (34)	50 (42)	9 (24)	11 (52)	287 (38)
Widow(er)	4 (6)	0	35 (7)	5 (4)	2 (6)	0	46 (6)
Employment *N* (%)							
Not working	13 (18)	7 (50)	123 (24)	64 (54)	3 (9)	12 (57)	222 (29)
Paid work	28 (39)	7 (50)	198 (39)	42 (35)	19 (58)	9 (43)	303 (40)
Retired	14 (20)	0	140 (28)	4 (3)	10 (30)	0	168 (22)
Unpaid work	16 (23)	0	44 (9)	9 (8)	1 (3)	0	70 (9)

*SD* standard deviation.

### Overview of Participants’ Responses and ICF Categories

The study yielded a total of 7652 meaningful concepts, which could be linked to 148 distinct categories according to the ICF. Among these, 9% (or 649 linked items in 41 categories) pertained to body functions and structures, 49% (3752 items in 77 categories) to activities and participation and 42% (3146 items in 28 categories) to environmental factors

The distribution of ICF chapters across the different WHO regions shows significant regional differences in the reporting of functioning domains. Percentages represent the proportion of mentions within each region, rather than the total sample, to highlight the relative importance of specific domains in different cultural contexts. This approach was chosen because it allows meaningful comparison across regions with varying sample sizes. Within the domain of ‘*Body Functions’*, the chapter *Mental functions* was most frequently reported in Europe (*N* = 236; 7%) and Southeast Asia (*N* = 76; 8%), while this category was completely absent in the Africa and Eastern Mediterranean regions. *Sensory functions and pain* were mainly reported in Southeast Asia (*N* = 56; 6%), the Americas (*N* = 26; 2%) and Europe (*N* = 82, 2%), with minimal mention in Africa (*N* = 3; 1%). Other body functions, such as cardiovascular, digestive and neuromusculoskeletal functions, appeared only sporadically and each represented less than 1% of the reports.

Within the domain of ‘*Activity and Participation’*, the chapter *Learning and applying knowledge* was widely reported across all regions, with the highest percentages in the Western Pacific (*N* = 63; 10%) and Africa (*N* = 47; 9%). Europe reported the highest absolute number (*N* = 163), but with a relatively lower percentage (5%). The chapter *General tasks and demands* was notably underreported in all regions (*N* = 1–2 and percentages of 0%). *Communication* was consistently reported across all regions, with the highest absolute numbers in Europe (*N* = 119; 4%) and the Americas (*N* = 89; 6%). *Mobility* was the most reported life domain within this chapter, where Europe (*N* = 314; 9%), the Americas (*N* = 251; 17%) and the Western Pacific (*N* = 120; 18%) showed high numbers. *Self-care* was moderately reported, with the highest numbers in Europe (*N* = 148; 4%) and Southeast Asia (*N* = 45; 5%). *Domestic life* was relatively evenly reported across all regions, with a peak in Europe (*N* = 91; 3%). This chapter appeared to be widely recognised as relevant for participation in daily life. The chapter *Interpersonal interactions and relationships* was reported less often. Only the Americas (*N* = 7; 0%) and Africa (*N* = 3; 1%) showed any mention. *Major life areas* was widely reported, with high relative numbers in Europe (*N* = 202; 6%), the Americas (*N* = 151; 10%) and the Western Pacific (*N* = 78; 12%). Percentages ranged from 6 to 13%, indicating broad recognition of work, education and economic participation as core aspects of functioning. *Community, social and civic life* also showed high numbers, especially in Europe (*N* = 402; 12%) and the Americas (*N* = 151; 10%). This chapter reflects the degree of social integration and participation in public life.

Within the domain of ’*Environmental Factors’*, there was a varied pattern across the five chapters. The chapter *Products and technology* was most often reported in Europe (*N* = 543; 16%) and the Americas (*N* = 233; 16%), followed by the Western Pacific (*N* = 53; 8%). The other regions, including Africa (*N* = 34; 7%) and Southeast Asia (*N* = 42; 5%), showed lower percentages. *Support and relationships* was the most prominently reported chapter within this domain. All regions showed relatively high percentages, with a peak in Africa (*N* = 154; 30%). Percentages ranged from 20% to 30%. The chapter *Attitudes* was only minimally reported, with the highest value in the Western Pacific (*N* = 21; 3%). In other regions, percentages remained below 0.02%. The chapter *Services, systems and policies* was most often reported in Europe (*N* = 159; 5%) and Southeast Asia (*N* = 78; 8%). In the Eastern Mediterranean and Western Pacific, data on this chapter were completely absent (Table 2).

**Table 2:** Overview of linked items to the different International Classification of Functioning (ICF) chapters per World Health Organization (WHO) Region [*N* (%)].

		Americas *N* = 1467	Eastern Mediterranean *N* = 561	Europe *N* = 3353	Southeast Asia *N* = 926	Western Pacific *N* = 652	Africa *N* = 517	Total *N* = 7476
Body Functions	Chapter 1: Mental functions	29 (0.02)	1 (0.00)	236 (0.07)	76 (0.08)	15 (0.02)	3 (0.01)	360 (4.71)
	Chapter 2: Sensory functions and pain	26 (0.02)	4 (0.01)	82 (0.02)	56 (0.06)	6 (0.01)	1 (0.00)	175 (2.29)
	Chapter 4: Functions of the cardiovascular, haematological and respiratory system	0	0	32 (0.01)	0	0	0	32 (0.42)
	Chapter 5: Functions of the digestive, metabolic and endocrine system	1 (0.00)	0	1 (0.00)	0	0	0	2 (0.03)
	Chapter 7: Neuromusculoskeletal and movement related functions	4 (0.00)	0	3 (0.00)	0	2 (0.00)	0	9 (0.12)
Activity & Participation	Chapter 1: Learning and applying knowledge	104 (0.07)	39 (0.07)	163 (0.05)	52 (0.06)	63 (0.10)	47 (0.09)	468 (6.13)
	Chapter 2: General tasks and demands	1 (0.00)	2 (0.00)	16 (0.00)	2 (0.00)	2 (0.00)	2 (0.00)	25 (0.33)
	Chapter 3: Communication	89 (0.06)	63 (0.11)	119 (0.04)	53 (0.06)	27 (0.04)	21 (0.04)	372 (4.87)
	Chapter 4: Mobility	251 (0.17)	98 (0.17)	314 (0.09)	109 (0.12)	120 (0.18)	84 (0.16)	976 (12.78)
	Chapter 5: Self Care	30 (0.02)	8 (0.01)	148 (0.04)	45 (0.05)	14 (0.02)	12 (0.02)	257 (3.37)
	Chapter 6: Domestic Life	48 (0.03)	41 (0.07)	91 (0.03)	32 (0.03)	33 (0.05)	41 (0.08)	286 (3.75)
	Chapter 7: Interpersonal interactions and relationships	7 (0.00)	0	41 (0.01)	4 (0.00)	1 (0.00)	3 (0.01)	56 (0.73)
	Chapter 8: Major life areas	89 (0.06)	74 (0.13)	202 (0.06)	72 (0.08)	78 (0.12)	52 (0.10)	567 (7.43)
	Chapter 9: Community, social and civic life	151 (0.10)	43 (0.08)	402 (0.12)	69 (0.07)	41 (0.06)	39 (0.08)	745 (9.76)
Environmental Factors	Chapter 1: Products and technology	233 (0.16)	32 (0.06)	543 (0.16)	42 (0.05)	53 (0.08)	34 (0.07)	937 (12.27)
	Chapter 2: Natural environment and human made changes to environment	24 (0.02)	0	37 (0.01)	6 (0.01)	9 (0.01)	4 (0.01)	80 (1.05)
	Chapter 3: Support and relationships	296 (0.20)	144 (0.26)	740 (0.22)	221 (0.24)	166(0.25)	154 (0.30)	1,721 (22.54)
	Chapter 4: Attitudes	32 (0.02)	12 (0.02)	24 (0.01)	9 (0.01)	21 (0.03)	5 (0.01)	103 (1.35)
	Chapter 5: Services, systems and policies	52 (0.04)	0	159 (0.05)	78 (0.08)	1 (0.00)	15 (0.03)	305 (3.99)

Percentages represent the proportion of mentions within each WHO region.

Within the component *body functions* (b), the top five identified second-level codes were emotional functions (b152; *n* = 132), temperament and personality functions (b126; *n* = 95), seeing functions (b210; *n* = 52), memory functions (b144; *n* = 40) and sensation of pain (b280; *n* = 38) (Table 3). No items were assigned to chapters 3, voice and speech functions and 6, genitourinary and reproductive functions of the ICF (Table 2). The largest group of ICF-linked codes appeared in Chapter 1, with a significant focus on confidence (B1266; *n* = 21) and energy level (B1300; *n* = 24) as crucial factors for functioning with vision loss. Additionally, anxiety, sadness and depressive symptoms were frequently mentioned by participants.

**Table 3:** Overview of the absolute frequencies of the number of times the item is mentioned in the International Classification of Functioning (ICF) Category Body Function (b).

Second-level category		*N* = 578
b114	Orientation functions	7 (0.01)
b126	Temperament and personality functions	95 (0.16)
b130	Energy and drive functions	37 (0.06)
b140	Attention functions	12 (0.02)
b144	Memory functions	40 (0.07)
b152	Emotional functions	132 (0.23)
b156	Perceptual functions	4 (0.01)
b160	Thought functions	3 (0.01)
b164	Higher-level cognitive functions	30 (0.05)
b210	Seeing functions	52 (0.09)
b230	Hearing functions	29 (0.05)
b235	Vestibular functions	21 (0.04)
b240	Sensation associated with hearing and vestibular function	35 (0.06)
b280	Sensation of pain	38 (0.07)
b410	Heart functions	2 (0.00)
b420	Blood pressure functions	2 (0.00)
b440	Respiration functions	28 (0.05)
b530	Weight maintenance functions	2 (0.00)
b710	Mobility of joint functions	3 (0.01)
b755	Involuntary movement reaction functions	1 (0.00)
b760	Control of voluntary movement functions	3 (0.01)
b780	Sensations related to muscles and movement functions	2 (0.00)

For *body structures* (s), only three second-level codes were used: structure of head and neck region (s710; *n* = 35), structure of shoulder region (s720; *n* = 14) and structure of the trunk (s760; *n* = 22). These findings mainly relate to pain resulting from postural challenges. Persons with vision loss often walk more bent forward or adopt a stiffer posture out of fear of bumping into obstacles. Such compensatory strategies can lead to back and neck pain, as well as increased tension in the shoulder and trunk regions.

The largest group of linked items was assigned to the category of *activity and participation* (d). The top five identified second-level codes within this category were recreation and leisure (d920; *n* = 671), using transportation (d470; *n* = 385), remunerative employment (d850; *n* = 328), moving around using equipment (d465; *n* = 215) and communicating with receiving nonverbal messages (d315; *n* = 211). The issues related to receiving nonverbal messages primarily stemmed from difficulties in recognising faces, body language, eye contact and traffic signs during mobility as reported in the surveys and interviews (Table 4).

**Table 4:** Overview of the absolute frequencies of the number of times the item is mentioned in the International Classification of Functioning (ICF) Category Activity & Participation (d).

Second-level category		*N* = 3755
d100	Watching	87 (0.02)
d115	Listening	142 (0.04)
d160	Focusing attention	16 (0.00)
d163	Thinking	13 (0.00)
d166	Reading	177 (0.05)
d170	Writing	16 (0.00)
d175	Solving problems	17 (0.00)
d240	Handling stress and other psychological demands	25 (O.01)
d310	Communicating with—receiving—spoken messages	108 (0.03)
d315	Communicating with—receiving—nonverbal messages	211 (0.06)
d330	Speaking	6 (0.00)
d340	Producing messages in formal sign language	30 (0.01)
d360	Using communication devices and techniques	9 (0.00)
d399	Communication, unspecified	8 (0.00)
d450	Walking	193 (0.05)
d455	Moving around	13 (0.00)
d460	Moving around in different locations	95 (0.03)
d465	Moving around using equipment	215 (0.06)
d470	Using transportation	385 (0.10)
d475	Driving	75 (0.02)
d520	Caring body parts	138 (0.04)
d540	Dressing	58 (0.02)
d560	Drinking	2 (0.00)
d570	Looking after one’s health	49 (0.01)
d599	Self-care, unspecified	10 (0.00)
d620	Acquisition of goods and services	119 (0.03)
d630	Preparing meals	61 (0.02)
d640	Doing housework	76 (0.02)
d650	Caring for household objects	30 (0.01)
d710	Basic interpersonal relations	12 (0.00)
d720	Complex interpersonal relations	2 (0.00)
d740	Formal relationships	14 (0.00)
d750	Informal social relationship	22 (0.01)
d770	Intimate relationship	6 (0.00)
d810	Informal education	38 (0.01)
d820	School education	2 (0.00)
d830	Higher education	15 (0.00)
d839	Education unspecified	4 (0.00)
d845	Acquiring, keeping and terminating a job	78 (0.02)
d850	Remunerative employment	328 (0.09)
d855	Non-remunerative employment	95 (0.03)
d860	Basic economic transaction	7 (0.00)
d910	Community life	47 (0.01)
d920	Recreation and leisure	671 (0.18)
d930	Religion and spirituality	11 (0.00)
d940	Human rights	10 (0.00)
d950	Political life and citizenship	9 (0.00)

The *environmental factors* (e), which represent the second largest group of linked meaningful concepts, the top five identified second-level codes were immediate family (e310; *n* = 737), products and technology for personal use in daily living (e115; *n* = 698), which included both small daily living aids and smartphones. Additionally, friends (e320; *n* = 290), extended family (e315; *n* = 236) and products and technology for communication (e125; *n* = 204) were reported to be important (Table 5).

**Table 5:** Overview of the absolute frequencies of the number of times the item is mentioned in the International Classification of Functioning (ICF) Category Environmental Factors (e).

Second-level category		*N* = 3146
e115	Products and technology for personal use in daily living	698 (0.22)
e120	Products and technology for personal indoor and outdoor mobility and transportation	35 (0.01)
e125	Products and technology for communication	204 (0.06)
e235	Human-caused events	36 (0.01)
e240	Light	44 (0.01)
e310	Immediate family	737 (0.23)
e315	Extended family	236 (0.08)
e320	Friends	290 (0.09)
e325	Acquaintances, peers, colleagues, neighbours and community members	66 (0.02)
e330	People in positions of authority	11 (0.00)
e340	Personal care providers and personal assistants	83 (0.03)
e345	Strangers	75 (0.02)
e350	Domesticated animals	37 (0.01)
e355	Health professionals	41 (0.01)
e360	Other professionals	29 (0.01)
e399	Support and relationships, unspecified	116 (0.04)
e410	Individual attitudes of immediate family members	2 (0.00)
e425	Individual attitudes of acquaintances, peers, colleagues, neighbours and community members	3 (0.00)
e460	Societal attitudes	82 (0.03)
e465	Social norms, practices and ideologies	8 (0.00)
e499	Attitudes, other specified	8 (0.00)
e520	Open space planning services, systems and policies	3 (0.00)
e535	Communication services, systems and policies	7 (0.00)
e550	Legal services, systems and policies	64 (0.02)
e560	Media services, systems and policies	71 (0.02)
e565	Economic services, systems and policies	11 (0.00)
e580	Health services, systems and policies	63 (0.02)
e599	Services, systems and policies, unspecified	86 (0.03)

## Discussion

The study offered a comprehensive analysis of the impact of vision loss, mapped across various ICF categories and investigated the perspectives of individuals with vision loss on their functioning from a global perspective. By including a diverse sample of participants, this study aimed to provide a broad understanding of the challenges and perceptions related to functioning and disability among people across the WHO regions in 61 countries.

### Body Functions

Within the ‘body functions’ domain, participants frequently reported feelings of depression and other emotional challenges, requiring more time to complete tasks compared to individuals with normal vision and experiencing significant fatigue. This aligns with previous research indicating that individuals with vision loss often face additional (mental) health problems such as anxiety and depression [15], as well as fatigue [39]. Schakel et al. [40] found that individuals with visual impairment experienced higher levels of fatigue compared to those without such impairment. This severe fatigue may result from the considerable mental effort required to compensate for vision loss, leading to an increased cognitive load as individuals adapt their daily routines. This suggests that the cognitive challenges associated with vision loss extend beyond the physical aspects, shedding light on the mental and perceptual adaptations individuals undergo, which may contribute to elevated levels of fatigue in this population [41]. Europe and Southeast Asia placed relatively greater emphasis on ‘Body Functions’, particularly on mental health aspects. This may reflect heightened awareness of mental health issues or the presence of more advanced diagnostic infrastructures in these regions.

In this study, only limited attention was given to seeing functions (b210) from the perspective of the lived experience of persons with vision loss This may be explained by the fact that participants regarded vision loss as an obvious underlying condition and therefore focused primarily on its consequences such as limitations in mobility, communication and emotional well-being, rather than explicitly mentioning the impairment itself. This contrasts with our preparatory study, which examined the perspective of healthcare professionals, where seeing functions were emphasised much more strongly [42]. A plausible explanation is that professionals typically adopt a biomedical perspective and prioritise the primary sensory deficit, whereas persons with vision loss tend to highlight the functional and psychosocial impact on daily life. These differing perspectives underscore the importance of combining both viewpoints when developing an ICF Core Set, as they reflect complementary dimensions of functioning.

### Activities and Participation

Within the domain of ‘Activities and Participation’, the results indicate a pronounced emphasis on the life domains mobility, communication and community, social and civic life. These areas are consistently reported across nearly all WHO regions, underscoring their universal relevance in assessing human functioning. In contrast, domains such as interpersonal relationships and general tasks and demands remain notably underrepresented. The Americas and the Eastern Mediterranean reported more extensively on ‘Activities and Participation’.

This may point to gaps in data collection or reflect cultural differences in how participation is conceptualised. The high reporting of mobility and self-care in Europe and the Americas may be linked to greater availability of rehabilitation and healthcare services [43]. Meanwhile, the broader distribution of data on learning and communication suggests a globally shared recognition of the importance of cognitive and social competencies. Mobility, in particular, plays a crucial role in supporting the independence of individuals, especially those with visual impairments, as it enables navigation within both domestic and public environments.

The need for social interaction is driven significantly by the lack of social support in the surroundings of persons with visual impairment, indicating potential challenges within their social environments [44]. This highlights the critical role of familial connections in providing essential social support and enhancing the social well-being of individuals dealing with visual impairment [45]. This research supports these findings, with the network of family and friends that emerged as vital for participants.

The present results are consistent with previous studies, showing that most participants were unemployed or underemployed [46, 47]. According to Daniëls and colleagues [48], factors such as the level of social support, dependency on others and the ability to use assistive devices are significant predictors of employment status. This underscores the importance of a supportive social network and access to appropriate assistive technologies in facilitating employment opportunities for individuals with visual impairments. The environmental factors show that, in general and across all the individual WHO regions, the assistance of other people, technological aids, family and auditory aids were mentioned frequently, which indicates that these themes are important. The assistance of other people was an important theme that was observed in this study. In investigations that assessed external factors in general, the help of others to execute tasks was deemed important as well [10]. In studies that specifically focused on the area of education, it was also observed that the attitudes of parents, teachers and peers were important factors which determined whether a person with visual impairment could pursue an education [10, 49]. In the area of work, the attitudes of co-workers and employers had an important impact on the functioning of a person with visual impairment [50–52]. Also, in the area of mobility, the assistance of others, either to drive the person with a visual impairment to their destination personally or to guide them through other means of transport, played an important role [8]. Another one of the most frequently mentioned themes in the environmental factors chapter was family. Similar to the present study, other investigations found that family members are a crucial source of emotional support and/or of assistance in (daily) tasks [10, 53] and thus contribute essentially to the functioning of people with vision loss. However, family can also have a negative influence, for example, when the family does not possess the knowledge on how to assist a visually impaired person [54], is not involved with the visually impaired person due to lack of time [53, 54] or shows a lack of friendly or equal treatment [53]. In this study, immediate and extended family was often mentioned.

### Environmental Factors

A theme that was mentioned across many areas of life was technology. Assistive technologies are a key as they influence the ability to function in daily life. The combination of the codes ‘technological aids’ and ‘smartphones’ was mentioned 546 times, contributing to 11.2% of the total number of codes. There are several studies that focused specifically on technological assistive aids, which showed that those products are important to improve functioning and quality of life for people with VI [55]. Assistive technologies that are most frequently used involve receiving or giving information via an auditory medium, such as voice messages or auditory transcripts of texts [10, 55] or adapting the visual medium, such as contrast or colour change or magnification [10, 55]. Technological devices, particularly smartphones, are the most frequently used and beneficial products for participants. These devices significantly reduce the time required to access information and give access to a variety of apps, which convert the smartphone into a useful aid. Smartphones can play a pivotal role in empowering individuals with visual impairments, promoting inclusivity and improving overall quality of life.

During the development of the ICF Core Set Vision Loss, several preparatory studies were conducted. One of these involved conducting focus groups in Flanders, Belgium (a high-income country) to address the specific needs and challenges faced by individuals with vision loss. In contrast to our local focus group study [53], the current investigation reveals a greater proportion of linked concepts on a global scale, but with a slightly different distribution across the various components. In the focus group study, a total of 409 meaningful concepts were linked to the ICF, spread over 119 categories, of which 17% in the chapters ‘body functions and structures’, compared to 9% in the current study, 51% ‘activities and participation’, compared to 49% and 29% ‘environmental factors’, compared to 41%. The global perspective of the current study highlights the need to obtain a more comprehensive understanding of the impact of visual impairment on daily functioning when developing an ICF Core Set, in contrast to focusing on a high-income country alone.

### Strengths and Limitations

A strength of this study lies in its inclusion of a broad population of 763 individuals with blindness or visual impairment, covering 61 countries across all WHO regions. The large and diverse sample of blind and visually impaired individuals enhances the generalisability of the findings, reflecting a wide range of perspectives and experiences within the target population from a global perspective. The sample was well-distributed across various ages, sex, education levels and living and employment statuses. Nevertheless, the study faced limitations inherent to global research. Despite efforts to include participants from all WHO regions, the distribution was uneven, with a notable concentration of data from Europe, which may have affected the generalisability of the results. The underrepresentation of low-income countries, specifically in the Western Pacific and African regions, likely reflects both limited research engagement and challenges in motivating participation. Several structural and contextual factors contributed to the European predominance: greater internet access and digital literacy facilitated online survey completion, while recruitment through established research networks in Europe may have increased response rates. Although the survey was offered in eleven languages, these were more closely aligned with European populations, potentially restricting participation elsewhere. Finally, socio-economic constraints in low-income settings, including limited time, resources and institutional support, posed additional barriers to participation. To ensure the generalisability of the findings, it is essential to account for cultural differences in the subsequent preparatory phases of the ICF Core Set development. Cultural and contextual factors can significantly influence the perception of functioning and priorities reported by individuals with vision loss. Therefore, the consensus phase must guarantee equal representation of all WHO regions within the decision-making process. This balanced participation is critical to avoid regional bias and to develop a globally applicable Core Set that reflects diverse experiences and needs across different health systems and sociocultural contexts.

Additionally, the study intentionally excluded personal factors, as these are not defined within the ICF framework because of conceptual and taxonomic challenges when the ICF was developed and continue to be a topic for discussion in literature. Personal factors are considered to be controversial, as they are often viewed as ‘too personal’ (e.g., motivation and coping style) and should therefore not be used to avoid the risk of stigmatisation, labelling [56] or blaming, which may suggest that the issue resides within a person [57]. Several efforts have been made to define personal factors in a more neutral way [58]. However, the current findings and interpretation clearly show their relevance. Cultural background, socio-economic status and demographic characteristics such as age and sex influenced participation and functioning in meaningful ways. This apparent contradiction reflects a limitation of the current ICF taxonomy rather than the irrelevance of these factors. Therefore, it should be emphasised that, despite their absence in the coding system, personal factors remain critical for understanding functioning, particularly in clinical and applied contexts. Future research and consensus-building should explore strategies to integrate these dimensions without compromising the neutrality and universality of the ICF.

## Conclusion

The study highlights the multifaceted challenges faced by individuals with vision loss, particularly in the domains of activities and participation, such as recreation, transportation and communication. Significant emotional and psychological factors, including confidence, energy levels, anxiety and depressive symptoms, were identified within the body functions category. Additionally, environmental factors, such as family support and assistive technology, were crucial. This comprehensive analysis provides a holistic view of the experiences and needs of those with vision loss, offering valuable insights for the development of targeted support systems and interventions. The findings emphasise the necessity for tailored approaches to address the diverse challenges encountered in various functional domains.

This study, which focuses on mapping the patient perspective, is part of a broader research initiative aimed at developing both a comprehensive and a brief ICF Core Set Vision Loss. It constitutes one of the preparatory phases, alongside studies exploring perspectives from research and healthcare professionals. The next stage in the ICF Core Set development process involves integrating and synthesising findings from all preparatory studies to formulate the final ICF Core Set Vision Loss.

## Data Availability

The datasets generated during and/or analysed during the current study are available from the corresponding author on reasonable request

## References

[1] Flaxman SR, Bourne RRA, Resnikoff S, Ackland P, Braithwaite T, Cicinelli MV, et al. Global causes of blindness and distance vision impairment 1990–2020: a systematic review and meta-analysis. Lancet Glob Health. 2017;5:e1221–34. 10.1016/S2214-109X(17)30393-5. 29032195 10.1016/S2214-109X(17)30393-5

[2] Bourne RRA, Steinmetz JD, Saylan M, Mersha AM, Weldemariam AH, Wondmeneh TG, et al. Causes of blindness and vision impairment in 2020 and trends over 30 years, and prevalence of avoidable blindness in relation to VISION 2020: the right to sight: an analysis for the Global Burden of Disease Study. Lancet Glob Health. 2021;9:e144–60. 10.1016/S2214-109X(20)30489-7. 33275949 10.1016/S2214-109X(20)30489-7 PMC7820391

[3] Bourne RRA, Flaxman SR, Braithwaite T, Cicinelli MV, Das A, Jonas JB, et al. Magnitude, temporal trends, and projections of the global prevalence of blindness and distance and near vision impairment: a systematic review and meta-analysis. Lancet Glob Health. 2017;5:e888–97. 10.1016/S2214-109X(17)30293-0. 28779882 10.1016/S2214-109X(17)30293-0

[4] Leissner J, Coenen M, Froehlich S, Loyola D, Cieza A. What explains health in persons with visual impairment?. Health Qual Life Outcomes. 2014;12:65 10.1186/1477-7525-12-65. 24886326 10.1186/1477-7525-12-65 PMC4020575

[5] Rulli E, Quaranta L, Riva I, Poli D, Hollander L, Galli F, et al. Visual field loss and vision-related quality of life in the Italian Primary Open Angle Glaucoma Study. Sci Rep. 2018;8:619. 10.1038/s41598-017-19113-z. 29330448 10.1038/s41598-017-19113-z PMC5766542

[6] Liu CJ, Chang MC. Interventions within the scope of occupational therapy practice to improve performance of daily activities for older adults with low vision: a systematic review. Am J Occup Ther. 2020;74:7401185010p1–18. 10.5014/ajot.2020.038372. 10.5014/ajot.2020.038372 PMC7018463 32078506

[7] Smallfield S, Berger S, Hillman B, Saltzgaber P, Giger J, Kaldenberg J. Living with low vision: strategies supporting daily activity. Occup Ther Health Care. 2017;31:312–28. 10.1080/07380577.2017.1384969. 29043887 10.1080/07380577.2017.1384969

[8] Douglas G, Pavey S, Corcoran C, Clements B. Evaluating the use of the ICF as a framework for interviewing people with a visual impairment about their mobility and travel. Br J Vis Impair. 2012;30:6–21. 10.1177/0264619611428932.

[9] Alma MA, Van Der Mei SF, Melis-Dankers BJM, Van Tilburg TG, Groothoff JW, Suurmeijer TPBM. Participation of the elderly after vision loss. Disabil Rehabil. 2010;33:63–72. 10.3109/09638288.2010.488711. 20518624 10.3109/09638288.2010.488711

[10] Salminen AL, Karhula ME. Young persons with visual impairment: challenges of participation. Scand J Occup Ther. 2014;21:267–76. 10.3109/11038128.2014.899622. 24784723 10.3109/11038128.2014.899622

[11] Yibekal BT, Alemu DS, Anbesse DH, Alemayehu AM, Alimaw YA. Vision-related quality of life among adult patients with visual impairment at University of Gondar, Northwest Ethiopia. J Ophthalmol. 2020;2020:1–7. 10.1155/2020/9056097. 10.1155/2020/9056097 PMC7125459 32280539

[12] Brunes A, Hansen MB, Heir T. Loneliness among adults with visual impairment: prevalence, associated factors, and relationship to life satisfaction. Health Qual Life Outcomes. 2019;17:24 10.1186/s12955-019-1096-y. 30709406 10.1186/s12955-019-1096-y PMC6359849

[13] Harrabi H, Aubin MJ, Zunzunegui MV, Haddad S, Freeman EE. Visual difficulty and employment status in the world. PLoS ONE. 2014;9:e88306 10.1371/journal.pone.0088306. 24516632 10.1371/journal.pone.0088306 PMC3917855

[14] Veerman L, Heppe E, Gold D, Kef S. Intra- and interpersonal factors in adolescence predicting loneliness among young adults with visual impairments. J Vis Impair Blind. 2019;113:7–18. 10.1177/0145482X18818615.

[15] van der Aa HPA, van Rens GHMB, Comijs HC, Margrain TH, Gallindo-Garre F, Twisk JWR, et al. Stepped care for depression and anxiety in visually impaired older adults: multicentre randomised controlled trial. BMJ. 2015;351:h6127. 26597263 10.1136/bmj.h6127 PMC4655616

[16] Schakel W, Bode C, Van De Ven PM, Van Der Aa HPA, Hulshof CTJ, Van Rens GHMB, et al. Understanding fatigue in adults with visual impairment: a path analysis study of sociodemographic, psychological and health-related factors. PLoS ONE. 2019;14:e0224340. 10.1371/journal.pone.0224340. 10.1371/journal.pone.0224340 PMC6814229 31652298

[17] Legood R, Scuffham P, Cryer C. Are we blind to injuries in the visually impaired? A review of the literature. Injury Prev. 2002;8:155–60. 10.1136/ip.8.2.155. 10.1136/ip.8.2.155 PMC1730864 12120837

[18] Lamoureux E, Gadgil S, Pesudovs K, Keeffe J, Fenwick E, Dirani M, et al. The relationship between visual function, duration and main causes of vision loss and falls in older people with low vision. Graefes Arch Clin Exp Ophthalmol. 2010;248:527–33. 10.1007/s00417-009-1260-x. 20054556 10.1007/s00417-009-1260-x

[19] De Boer MR, Pluijm SMF, Lips P, Moll AC, Völker-Dieben HJ, Deeg DJH, et al. Different aspects of visual impairment as risk factors for falls and fractures in older men and women. J Bone and Miner Res. 2004;19:1539–47. 10.1359/JBMR.040504. 15312256 10.1359/JBMR.040504

[20] Hong T, Mitchell P, Burlutsky G, Samarawickrama C, Wang JJ. Visual impairment and the incidence of falls and fractures among older people: longitudinal findings from the Blue Mountains Eye Study. Invest Ophthalmol Vis Sci. 2014;55:7589–93. 10.1167/iovs.14-14262. 25370514 10.1167/iovs.14-14262

[21] Yen TH, Liou TH, Chang KH, Wu NN, Chou LC, Chen HC. Systematic review of ICF core set from 2001 to 2012. Disabil Rehabil Inf Healthc. 2014;36:177–84. 10.3109/09638288.2013.782359. 10.3109/09638288.2013.782359 23651126

[22] Karlsson E, Gustafsson J. Validation of the International Classification of Functioning, Disability and Health (ICF) Core Sets from 2001 to 2019—a scoping review. Disabil Rehabil. 2021;44:3736–48. 10.1080/09638288.2021.1878562. 10.1080/09638288.2021.1878562 33535017

[23] Selb M, Escorpizo R, Kostanjsek N, Stucki G, Üstün B, Cieza A. A guide on how to develop an international classification of functioning, disability and health core set. Eur J Phys Rehabil Med. 2015;51:105–17. 24686893

[24] Granberg S. Functioning and disability in adults with hearing loss. Preparatory studies in the ICF Core Sets for Hearing Loss project. 2015. Available from: https://www.researchgate.net/publication/281741882. Accessed 14 Feb 2026.

[25] Wittich W, Dumassais S, Jaiswal A, Ogedengbe TO, Lopez R, Granberg S. 2025 [cited 2025 Apr 20]. The WHO ICF Core Set for Deafblindness: the comprehensive core set. Available from: https://osf.io/a942k/.

[26] Wittich W, Dumassais S, Jaiswal A, Ogedengbe TO, Lopez R, Granberg S. 2025 [cited 2025 Apr 20]. The WHO ICF Core Set for Deafblindness: the brief core set. Available from: https://osf.io/nk29j/.

[27] Billiet L, Van de Velde D, Overbury O, Van Nispen RM. International classification of functioning, disability and health core set for vision loss: a discussion paper and invitation. Br J Vis Impair. 2022;40:109–16. 10.1177/02646196211055954.

[28] Billiet L, Van de Velde D, van der Aa HPA, De Baets S, van Nispen RMA. The development of an International Classification of Functioning, Disability, and Health (ICF) Core Set: a systematic review from 2001 to 2022. Ophthalmic Physiol Opt. 2024;44:413–25. 10.1111/opo.13269. 10.1111/opo.13269 PMC12872645 38251457

[29] Billiet L, Van de Velde D, van der Aa HPA, van Nispen RMA, De Baets S. How does vision loss affect daily living? A mixed method study in Flanders. Manuscript submitted for publication.

[30] WHO. Countries by WHO Region 2024 [cited 2024 Dec 26]. Available from: https://www.who.int/countries/.

[31] Christensen LB, Johnson RB, Turner LA. Research methods, design, and analysis. 12th ed. Essex: Pearson Education Limited; 2015.

[32] World Health Organization. Blindness and vision impairment [cited 2022 Dec 26]. Available from: https://www.who.int/news-room/fact-sheets/detail/blindness-and-visual-impairment#:~:text=Mild%20%E2%80%93%20visual%20acuity%20worse%20than,acuity%20worse%20than%203%2F60.

[33] Glässel A, Coenen M, Kollerits B, Cieza A. Validation of the extended ICF core set for stroke from the patient perspective using focus groups. Disabil Rehabil. 2012;34:157–66. 10.3109/09638288.2011.593680. 21967046 10.3109/09638288.2011.593680

[34] Kirchberger I, Coenen M, Hierl FX, Dieterle C, Seissler J, Stucki G, et al. Validation of the International Classification of Functioning, Disability and Health (ICF) Core Set for diabetes mellitus from the patient perspective using focus groups. Diabet Med. 2009;26:700–7. 10.1111/j.1464-5491.2009.02762.x. Jul. 19573119 10.1111/j.1464-5491.2009.02762.x

[35] Coenen M, Cieza A, Stamm TA, Amann E, Kollerits B, Stucki G. Validation of the International Classification of Functioning, Disability and Health (ICF) Core Set for rheumatoid arthritis from the patient perspective using focus groups. 2006. Available from: http://arthritis-research.com/content/8/4/R84Thisarticleisonlineat; http://arthritis-research.com/content/8/4/R84. Accessed 14 Feb 2026. 10.1186/ar1956 PMC1779412 16684371

[36] Aiachini B, Cremascoli S, Escorpizo R, Pistarini C. Validation of the ICF core set for vocational rehabilitation from the perspective of patients with spinal cord injury using focus groups. Disabil Rehabil. 2016;38:337–45. 10.3109/09638288.2015.1041611. 25924018 10.3109/09638288.2015.1041611

[37] Braun V, Clarke V. Thematic analysis: a practical guide. London, UK: Sage; 2021. p. 1–376.

[38] Cieza A, Geyh S, Chatterji S, Kostanjsek N, Üstün B, Stucki G. ICF linking rules: an update based on lessons learned. J Rehabil Med. 2005;37:212–8. 10.1080/16501970510040263. 16024476 10.1080/16501970510040263

[39] Schakel W, Bode C, Elsman EBM, van der Aa HPA, de Vries R, van Rens GHMB, et al. The association between visual impairment and fatigue: a systematic review and meta-analysis of observational studies. Ophthalmic Physiol Opt. 2019;9:399–413. 10.1111/opo.12647. 10.1111/opo.12647 PMC6899802 31696537

[40] Schakel W, Bode C, Van Der Aa HPA, Hulshof CTJ, Bosmans JE, Van Rens GHMB, et al. Exploring the patient perspective of fatigue in adults with visual impairment: a qualitative study. BMJ Open. 2017;7:e015023. 10.1136/bmjopen-2016-015023. 10.1136/bmjopen-2016-015023 PMC5724118 28775181

[41] Bakker K, Steultjens E, Price L. The lived experiences of adults with a visual impairment who experience fatigue when performing daily activities. Br J Occup Ther. 2019;82:485–92. 10.1177/0308022619841491.

[42] Billiet L, De Baets S, van der Aa HPA, van Nispen RMA, Van de Velde D. Healthcare professionals’ perspective on disability and functioning of individuals living with vision loss. Manuscript submitted for publication.

[43] World Health Organization. World report on vision. Geneva: World Health Organization; 2019.

[44] Cimarolli VR, Boerner K. Social support and well-being in adults who are visually impaired. J Vis Impair Blind. 2005;99. Available from: http://www.afb.org/jvib/jvib990904.asp.

[45] Vučinić V, Gligorović M, Anđelković M. Leisure in persons with vision impairment. Res Dev Disabil. 2020;102:103673 10.1016/j.ridd.2020.103673. 32388041 10.1016/j.ridd.2020.103673

[46] Chai YX, Gan ATL, Fenwick EK, Sui AY, Tan BKJ, Quek DQY, et al. Relationship between vision impairment and employment. Br J Ophthalmol. 2023;107:361–6. 10.1136/bjophthalmol-2021-319655. 34656985 10.1136/bjophthalmol-2021-319655

[47] Marques AP, Macedo AF, Lima Ramos P, Moreno LH, Butt T, Rubin G, et al. Productivity losses and their explanatory factors amongst people with impaired vision. Ophthalmic Epidemiol. 2019;26:378–92. 10.1080/09286586.2019.1632904. 31280630 10.1080/09286586.2019.1632904

[48] Daniëls R, van Nispen RM, de Vries R, Donker-Cools BHPM, Schaafsma FG, Hoving JL. Predictors for work participation of people with visual impairments: a systematic review and meta-analysis. Ophthalmic Physiol Opt. 2023;43:1223–54. 10.1111/opo.13188. 10.1111/opo.13188 37449334

[49] Bishop D, Rhind DJA. Barriers and enablers for visually impaired students at a UK Higher Education Institution. Br J Vis Impair. 2011;29:177–95. 10.1177/0264619611415329.

[50] Dong S, Warner A, Mamboleo G, Guerette A, Zalles MZ. Barriers in accommodation process among individuals with visual impairments. J Rehabil. 2017;83:27–35.

[51] Coffey M, Coufopoulos A, Kinghorn K. Barriers to employment for visually impaired women. Int J Workplace Health Manag. 2014;7:171–85. 10.1108/IJWHM-06-2013-0022.

[52] Bengisu M, Izbırak G, Mackieh A. Work-related challenges for individuals who are visually impaired in Turkey. J Vis Impair Blind. 2008;102:284–94. 10.1177/0145482X0810200504.

[53] Datta P, Sabir F. Influence of the family on students with visual impairment. In: Baikady R, Sajid SM, Przeperski J, Nadesan V, Islam MR, Gao J, editors. The Palgrave handbook of global social problems. Cham: Palgrave Macmillan; 2021. 10.1007/978-3-030-68127-2_69-1.

[54] Philip J, Hussaindeen JR, Jacob N, Sethuraman S, Swaminathan M. Parental perception of facilitators and barriers to activity and participation in an integrated tele-rehabilitation model for children with cerebral visual impairment in South India—a virtual focus group discussion study. Indian J Ophthalmol. 2023;71:601–7. 10.4103/ijo.IJO_1670_22. 36727370 10.4103/ijo.IJO_1670_22 PMC10228909

[55] Senjam SS, Manna S, Bascaran C. Smartphones-based assistive technology: accessibility features and apps for people with visual impairment, and its usage, challenges, and usability testing. Clin Optom. 2021;13:311–22. 10.2147/OPTO.S336361. 10.2147/OPTO.S336361 PMC8636846 34866955

[56] Grotkamp SL, Cibis WM, Nüchtern EAM, Von Mittelstaedt G, Seger WKF. Personal factors in the international classification of functioning, disability and health: prospective evidence. Aust J Rehabil Couns. 2012;18:1–24. 10.1017/jrc.2012.4.

[57] Whiteneck G. Models of disability: past, present, and future. Workshop on disability in America. Washington, DC: National Academies Press; 2006. p. 50–66.

[58] Heerkens YF, De Brouwer CPM, Engels JA, Van Der Gulden JWJ, Kant I. Elaboration of the contextual factors of the ICF for Occupational Health Care. Work. 2017;57:187–204. 10.3233/WOR-172546. 28582939 10.3233/WOR-172546

